# Hepatoprotective and Antioxidant Activities of a Medicine-Food Homology Formula and Its *Lactiplantibacillus plantarum*-Fermented Product Against Acetaminophen-Induced Oxidative Stress in HepG2 Cells

**DOI:** 10.5812/ijpr-168131

**Published:** 2026-04-12

**Authors:** Ching-Hui Hu, Simon Anthony Kayombo, Chih Min Yang

**Affiliations:** 1 Department of Food Science and Biotechnology, National Chung Hsing University, Taichung, Taiwan; 2 International Doctoral Program in Agriculture, National Chung Hsing University, Taichung, Taiwan

**Keywords:** Acetaminophen, Fermentation, HepG2 Cells, *Lactiplantibacillus plantarum*, Medicine-Food Homology, Oxidative Damage

## Abstract

**Background:**

Acetaminophen (APAP) overdose depletes glutathione (GSH) and induces oxidative liver injury. The medicine-food homology formula (MFHF) is composed of *Chrysanthemi flos* (CF), *Cassiae semen* (CS), and *Lycii fructus* (LF) (3:20:10, w/w/w). However, the effects of MFHF and its *Lactiplantibacillus plantarum*-fermented products (MFHFF) on quality markers and activities remain unclear.

**Objectives:**

This study investigated the prebiotic potential of MFHF; examined the stability of chlorogenic acid and chrysophanol before and after fermentation; compared hepatoprotective effects between MFHF and MFHFF in APAP-treated HepG2 cells; evaluated antioxidant activities; and assessed intra-formula interactions (additive/synergistic) among CF, CS, and LF.

**Methods:**

Fermentations were performed with *L. plantarum* BCRC12251 in 0 - 5% (w/v) MFHF substrates. Viable counts and pH were used to assess prebiotic effects. The HPLC was used to determine the content of chlorogenic acid and chrysophanol before and after fermentation. Antioxidant activities were measured by DPPH and potassium ferricyanide reducing antioxidant power (PFRAP) assays. Hepatoprotection was evaluated in APAP-treated HepG2 cells using cell viability, intracellular GSH, and malondialdehyde (MDA) as endpoints. Intra-formula interactions were evaluated by comparing observed and expected values. Data were analyzed using independent-sample t-tests and one-way ANOVA followed by Duncan’s multiple range test (P < 0.05).

**Results:**

MFHF enhanced *L. plantarum* growth by 26-fold and retained phytochemical markers after fermentation. Both MFHF and MFHFF increased viability and GSH and decreased MDA in a dose-dependent manner in APAP-challenged HepG2 cells. MFHF and MFHFF showed comparable antioxidant activities. Viability and DPPH activity were synergistic, whereas GSH, MDA, and PFRAP showed additive effects.

**Conclusions:**

MFHF could act as a prebiotic for *L. plantarum*. Fermentation preserved MFHF’s chemical integrity and hepatoprotective efficacy. Both preparations demonstrated comparable antioxidant and cytoprotective properties, supporting their potential as multifunctional candidates against oxidative liver injury in vitro and warranting further in vivo validation.

## 1. Background

Acetaminophen (APAP, paracetamol) is one of the most commonly used over-the-counter analgesic and antipyretic agents. At supratherapeutic doses, it causes acute liver failure due to excessive formation of N-acetyl-p-benzoquinone imine (NAPQI), depletion of intracellular glutathione (GSH), oxidative stress, lipid peroxidation, and mitochondrial dysfunction (1). Consequently, safe natural candidates with antioxidant and hepatoprotective activities are of sustained interest for mitigating APAP-induced hepatotoxicity. Liquid-state fermentation is widely applied to modulate phytochemical profiles and bioactivity of herbal substrates (2). *Lactiplantibacillus plantarum* is widely used in food and as a probiotic and has been reported to confer hepatoprotection in vitro and in vivo (3). The medicine-food homology formula employed in this study, composed of *Chrysanthemi flos* (CF), *Cassiae semen* (CS), and *Lycii fructus* (LF) at a weight ratio of 3:20:10, was derived from longstanding folk practices and is consumed as a liver-supporting decoction (4). CF, CS, and LF have been associated with liver support and redox balance (5-7). Despite their traditional use, the combined efficacy of CF, CS, and LF has not been systematically evaluated, and whether *L. plantarum* fermentation preserves key quality markers or alters functional activities remains unclear.

## 2. Objective

This study aimed to evaluate the prebiotic potential of MFHF by quantifying* L. plantarum* growth; examine the stability of chlorogenic acid and chrysophanol after fermentation; compare the hepatoprotective effects of MFHF and MFHFF in APAP-treated HepG2 cells using cell viability, intracellular GSH, and malondialdehyde (MDA); assess antioxidant activities using DPPH and PFRAP assays; and evaluate intra-formula interactions among CF, CS, and LF.

## 3. Methods

### 3.1. Sources of MFH Herbs and Preparation of Decoction Extracts

Raw materials of CF, CS, and LF, authenticated by Ko Da Pharmaceutical Co., Ltd. (Taoyuan, Taiwan), were extracted twice with hot water (1:20 for CF; 1:10 for the others) for 1 h each. Extracts were filtered, combined, concentrated under reduced pressure, and lyophilized into powders.

### 3.2. Source, Activation, and Proliferation of Lactic Acid Bacteria

*Lactiplantibacillus plantarum* (BCRC12251) from BCRC (Hsinchu, Taiwan) was thawed and inoculated (200 µL) into 4 mL of sterilized MRS broth, incubated at 37°C for 24 h for activation, and then subcultured for 16 - 18 h to ensure proliferation.

### 3.3. Establishment of Lactiplantibacillus plantarum Growth Curve

Bacterial suspension at optical density (OD) 600 nm was adjusted to 1.0, serially diluted, and plated on MRS agar. Colonies were counted after 48 h of incubation at 37°C to establish the correlation between absorbance and viable cell counts.

### 3.4. Determination of Reducing Sugar Content

The reducing sugar content in MFHF was measured using the DNS method with a glucose standard curve (0.2 - 1.0 mg/mL). Samples were reacted with the DNS reagent at 100°C for 10 min, and absorbance was measured at 540 nm. Results were expressed as mg glucose equivalent per gram, with MFHF containing approximately 620 mg GE/g.

### 3.5. Fermentation

Fermentation was carried out by inoculating *L. plantarum* at 1 × 10^3^ CFU/mL into sterilized substrates with varying MFHF concentrations (0%, 2.5%, and 5%, w/v). The upper limit of 5% (w/v) was selected to avoid excessive viscosity that could interfere with mixing and microbial enumeration. Fermentation proceeded at 37°C for 24 - 120 h, and viable counts were assessed before and after fermentation. Three substrates were used: control (0% MFHF) with yeast extract (0.6 g) and glucose (6.4 g) in 200 mL; 2.5% MFHF medium containing yeast extract (0.6 g), reducing sugar (3.2 g), and glucose (3.2 g); and 5% MFHF medium with yeast extract (0.6 g) and reducing sugar (6.4 g) but no added glucose.

### 3.6. Cell Culture

Human hepatoblastoma HepG2 cells (BCRC RM60025) were obtained from BCRC and cultured in minimum essential medium (MEM) supplemented with 1.5 g/L sodium bicarbonate, 2 mM L-glutamine, 0.1 mM non-essential amino acids, 1 mM sodium pyruvate, 50 µg/mL penicillin-streptomycin, and 10% fetal bovine serum (FBS). Cells were incubated at 37°C with 5% CO_2_ in a humidified incubator.

### 3.7. Cell Viability Assay

Cells were seeded at 2 × 10^5^ cells/mL in 96-well plates and incubated for 24 h. The medium was replaced with test samples containing 0.1% FBS and incubated for another 24 or 48 h. Based on the cytotoxicity assessment presented in Section 4.3, concentrations up to 250 µg/mL did not significantly reduce cell viability; therefore, the range of 50 - 250 µg/mL was selected for subsequent protective effect evaluation. Cell viability was measured by adding 10 µL of 10% CCK-8 reagent per well, incubating for 1 h at 37°C, and reading absorbance at 450 nm. Cell viability was calculated as:


$$Cell\;viability\;\left(\%\right)\;=\;\frac{\left({OD}_{Sample}-{OD}_{Blank}\right)}{\left({OD}_{Control}-{OD}_{Blank}\right)}\times 100\%$$

### 3.8. Determination of Intracellular Glutathione Content and Malondialdehyde Level

Cells were seeded at 3 × 10^6^ cells/mL in flasks and incubated for 24 h. After replacing the medium with test samples containing 0.1% FBS, cells were incubated for 24 or 48 h. Cells were collected, sonicated in PBS (for GSH) or extraction buffer (for MDA), centrifuged at 10,000×g for 10 minutes, and the supernatants were analyzed. Protein concentration was measured by Bio-Rad assay with BSA standard. Intracellular GSH and MDA levels were quantified using commercial kits (Elabscience, Hubei, China).

### 3.9. DPPH Radical Scavenging Ability Assay

Sample solutions of CF, CS, LF, MFHF, and MFHFF extracts were prepared in distilled water. Each (0.5 mL) was mixed with 0.2 mM DPPH solution and incubated in the dark at room temperature for 30 minutes. Absorbance was measured at 517 nm, and the inhibition rate was calculated.

### 3.10. Potassium Ferricyanide Reducing Antioxidant Power Assay

Ascorbic acid standards (5 - 30 µg/mL) and samples were mixed with phosphate buffer (pH 6.6) and 1% potassium ferricyanide, incubated at 50°C for 20 minutes. After adding 10% trichloroacetic acid and centrifugation, the supernatant was mixed with distilled water and ferric chloride. Absorbance at 700 nm was measured, and the results were expressed as mg ascorbic acid equivalent (AAE) per gram sample.

### 3.11. HPLC Analysis of Chlorogenic Acid and Chrysophanol

The HPLC analysis was performed on a Hitachi L-2000 system with a diode array detector (Hitachi, Tokyo, Japan). Chlorogenic acid and chrysophanol standards (> 98% purity, Chemfaces, Hubei, China) were prepared in 70% methanol (35 and 40 μg/mL, respectively). For chlorogenic acid, a 0.25 g sample was ultrasonically extracted with 25 mL of 70% methanol for 40 min, filtered, and adjusted to volume. For chrysophanol, a 0.5 g sample was refluxed with 50 mL of methanol for 3 h; the extract was evaporated, hydrolyzed with 10% HCl, extracted with ethyl acetate, evaporated, and reconstituted in 25 mL of methanol. Separation was performed on an RP-18 column using acetonitrile (A) and 0.1% phosphoric acid with the following gradients: Chlorogenic acid, 10 - 18% A (0 - 11 min), 18 - 20% A (11 - 30 min), and 20% A (30 - 40 min); chrysophanol, 40% A (0 - 15 min), 40 - 90% A (15 - 30 min), and 90% A (30 - 40 min). Detection was at 348 nm (chlorogenic acid) and 254 nm (chrysophanol). Flow rate was 1.0 mL/min, and injection volume was 10 μL.

### 3.12. Statistical Analysis

Data are expressed as the mean ± standard deviation (SD) from three independent experiments. Statistical comparisons between two groups were performed using an independent-sample t-test. For comparisons among multiple groups, one-way analysis of variance (ANOVA) followed by Duncan’s multiple range test was used. Statistical analysis was conducted using SPSS Statistics software (Version 22.0.0, IBM SPSS, Chicago, USA). A P-value < 0.05 was considered statistically significant.

## 4. Result

### 4.1. Optimization of Fermentation Conditions for Lactiplantibacillus plantarum Growth Using Medicine-Food Homology Formula as Substrate

**Figure 1.**
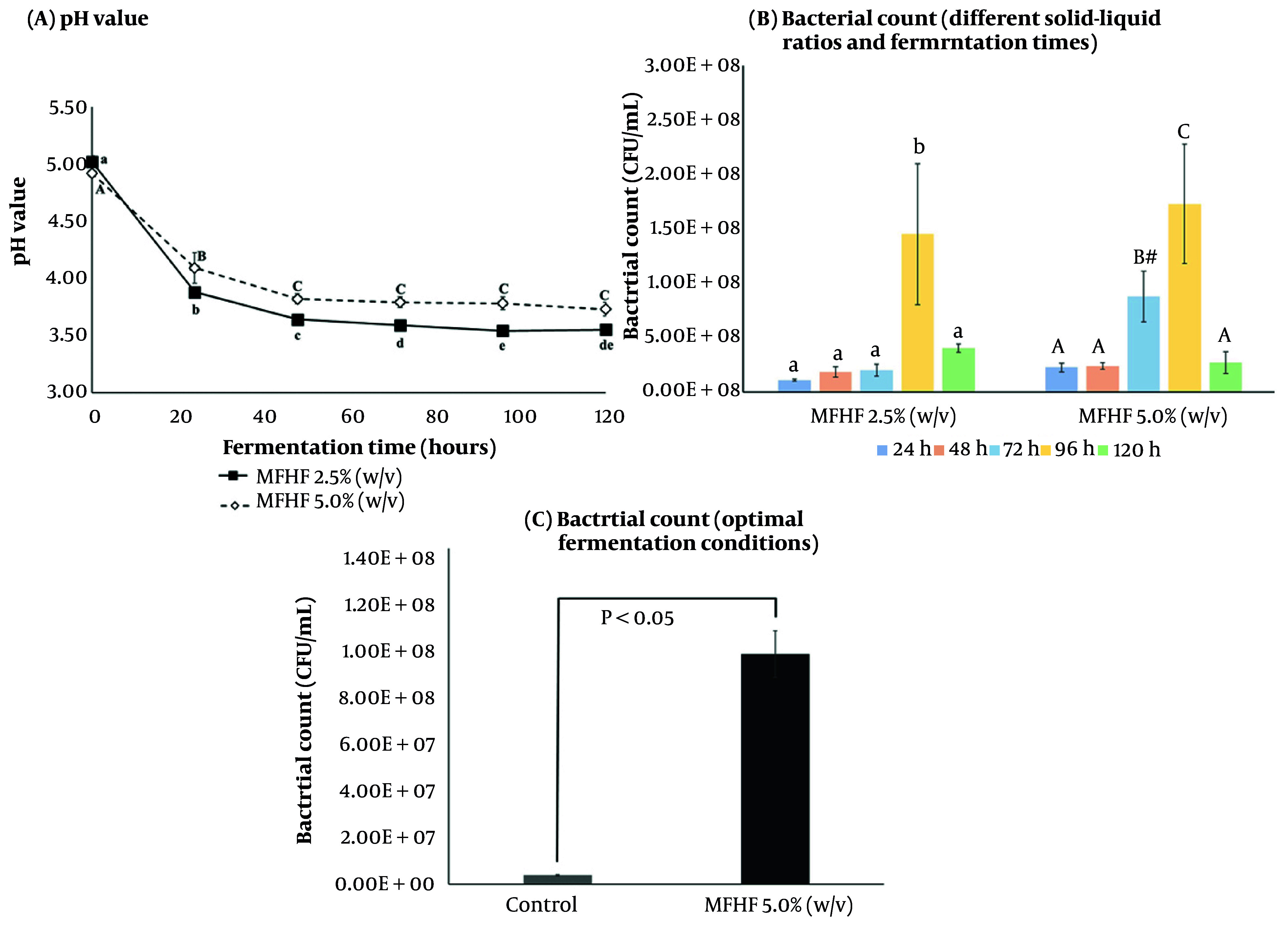
Effects of the medicine-food homology formula (MFHF) (2.5 and 5%, w/v) and fermentation durations (24 - 120 h) on: A, pH; and B, viable counts of *Lactiplantibacillus plantarum*; C, growth at 96 h in media with or without MFHF. Values are expressed as mean ± SD (n = 3). Different lowercase and uppercase letters indicate significant differences within the 2.5% (w/v) and 5% (w/v) groups, respectively (P < 0.05) (# represents a significant difference in bacterial counts between the 2.5% (w/v) and 5% (w/v) groups at 72 h (P < 0.05); A P < 0.05 is considered statistically significant).

A growth curve demonstrated a strong correlation between absorbance and viable counts (R² = 0.9993, data not shown). The inoculation level was 1.49 × 10^3^ CFU/mL. Initial pH values of 2.5% and 5.0% MFHF substrates were 5.02 and 4.9, respectively, declining sharply within 24 h and stabilizing through 120 h (Figure 1A). The 5.0% MFHF group showed the highest bacterial counts, peaking at 96 h (Figure 1B). Compared with the control with equal reducing sugar, growth in 5.0% MFHF was 26-fold higher at 96 h (Figure 1C). 

### 4.2. Chlorogenic Acid and Chrysophanol Content in Medicine-Food Homology Formula Before and After Fermentation

**Figure 2.**
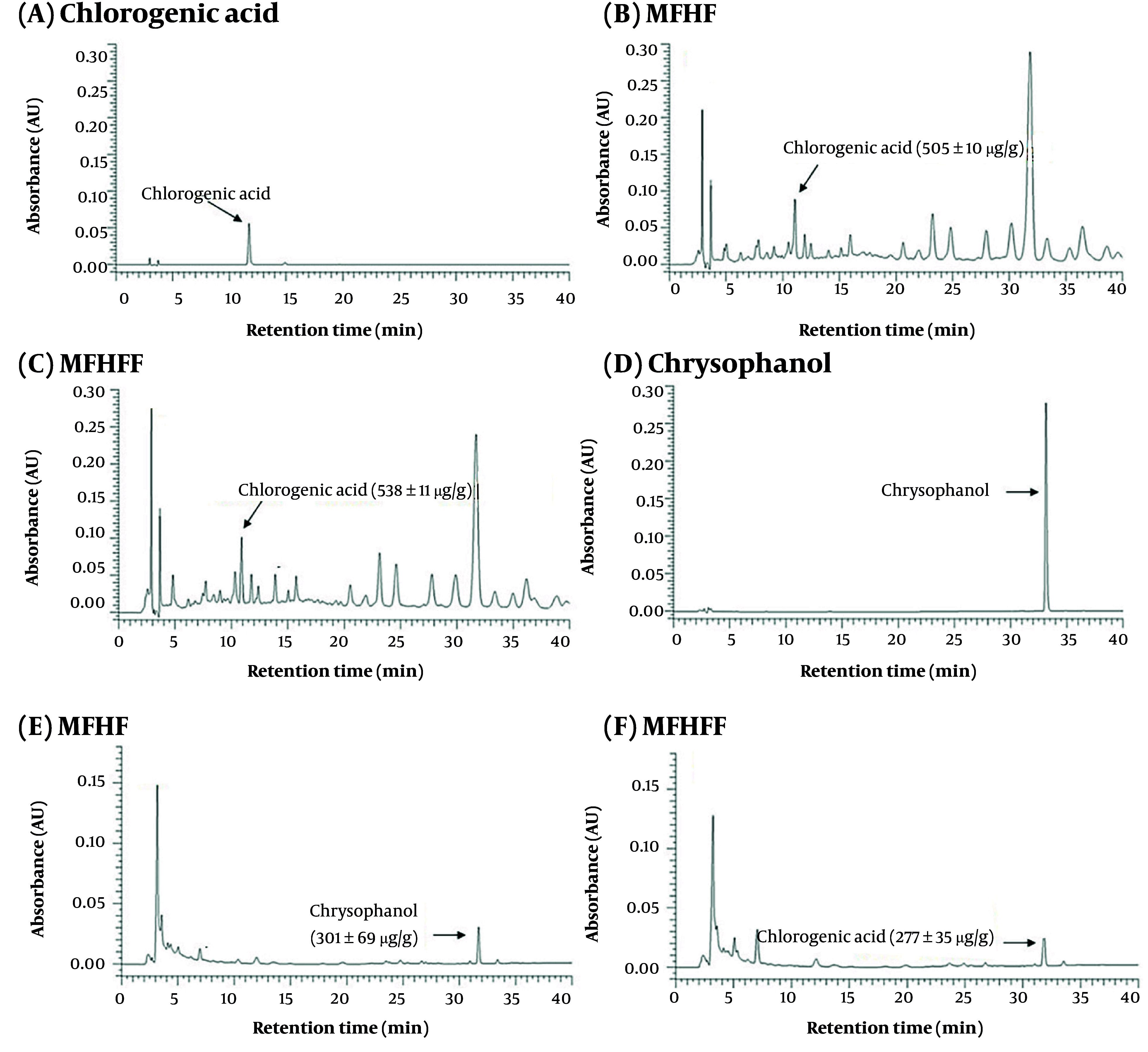
The HPLC profiles of A, medicine-food homology formula (MFHF); and B, its *Lactiplantibacillus plantarum*-fermented products (MFHFF) under the chromatographic conditions for chlorogenic acid. The HPLC profiles of C, MFHF; and D, MFHFF under the chromatographic conditions for chrysophanol.

Chlorogenic acid and chrysophanol, key markers for CF and CS quality control according to the Taiwan Herbal Pharmacopoeia, were detected in MFHF and MFHFF by HPLC, eluting at 11.7 and 33.1 min, respectively (Figure 2). Quantitative analysis showed no significant differences in their contents before and after fermentation (Figure 2). Notably, a peak at 14 min increased 3.5-fold. Another peak at 7 min rose 2.7-fold post-fermentation (Figure 2). 

### 4.3. Protective Effects of Medicine-Food Homology Formula and Lactiplantibacillus plantarum-Fermented Products on Cell Viability and Intracellular Glutathione and Malondialdehyde Levels in APAP-Treated HepG2 Cells

**Figure 3.**
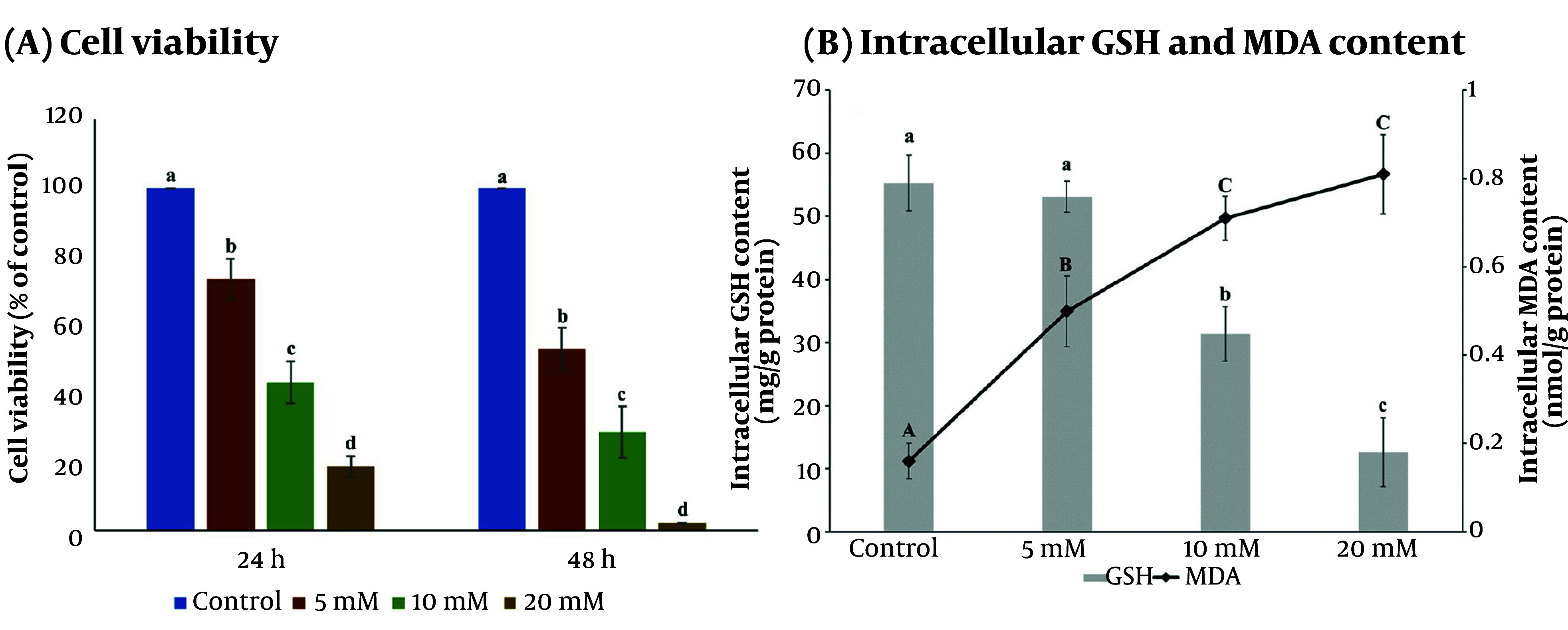
Effects of acetaminophen (APAP) on A, cell viability and intracellular; B, GSH; and MDA levels in HepG2 cells. Values are expressed as mean ± SD (n = 3). Different lowercase letters in cell viability at 24 h and 48 h of incubation indicate significant differences (P < 0.05). Different lowercase and uppercase letters indicate significant differences in GSH and MDA levels, respectively (P < 0.05).

**Figure 4.**
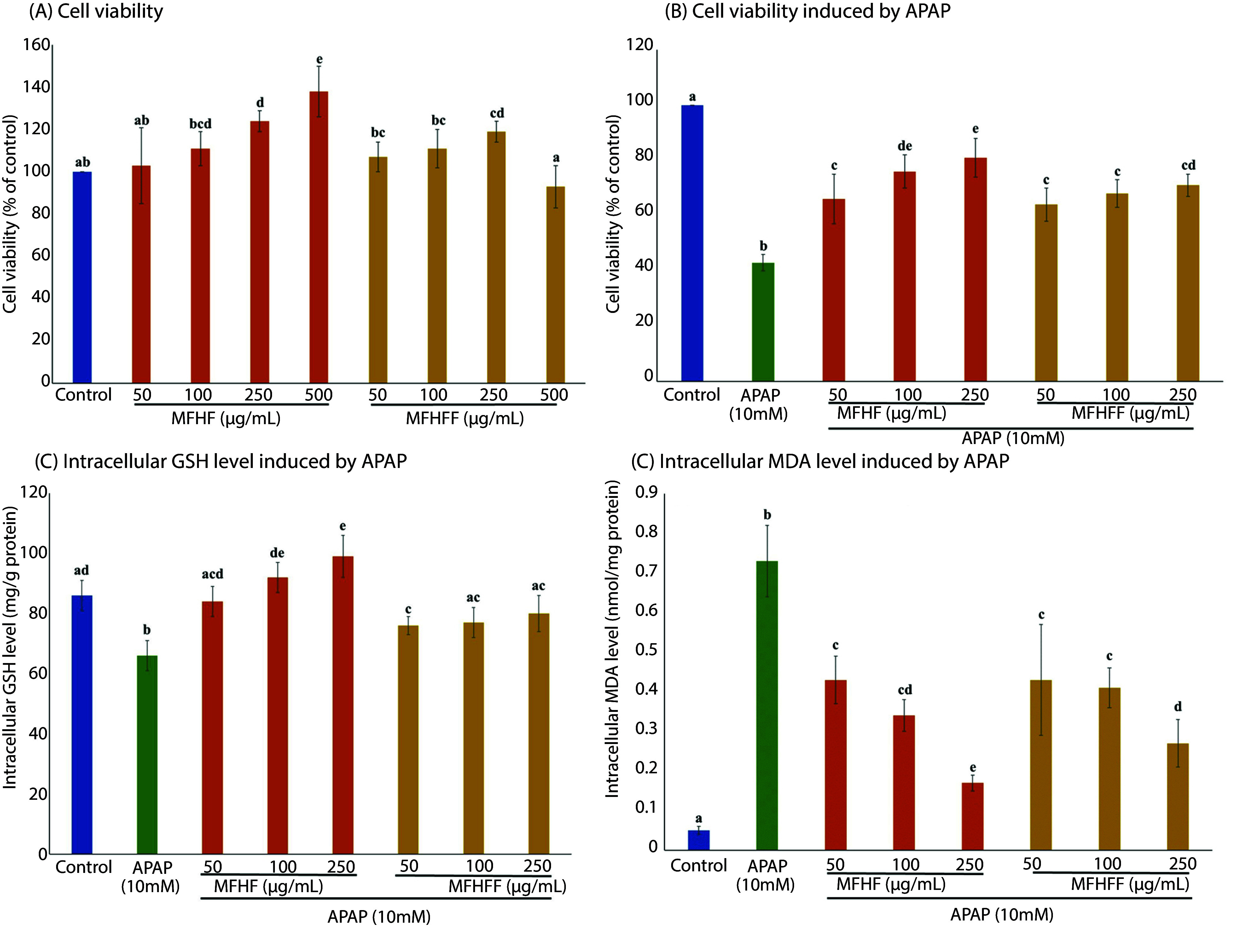
A, effects of the medicine-food homology formula (MFHF) and its *Lactiplantibacillus plantarum*-fermented products (MFHFF) on cell viability in HepG2 cells. Effects of MFHF and MFHFF on B, cell viability; C, intracellular glutathione (GSH); and D, malondialdehyde (MDA) levels induced by acetaminophen (APAP) in HepG2 cells. Values are expressed as mean ± SD (n = 3), with different lowercase letters indicating significant differences (P < 0.05).

The APAP treatment (5, 10, 20 mM) reduced HepG2 cell viability and altered intracellular GSH (decreased) and MDA (increased) in a concentration- and time-dependent manner (Figure 3). A 10 mM APAP exposure for 24 h was used as the oxidative damage model. The MFHF and MFHFF (50 - 250 µg/mL) showed no cytotoxicity (Figure 4A). APAP pretreatment for 24 h followed by co-treatment with MFHF or MFHFF for 24 h improved these parameters in a concentration-dependent manner: At 250 µg/mL, cell viability rose by 37% and 27%, GSH by 50% and 21%, and MDA decreased by 77% and 63%, respectively, compared with the APAP group (Figures 4B - D). MFHF showed synergistic or additive effects compared with the individual herbs, increasing cell viability by 103% (synergy score 1.21), GSH by 77% (synergy score 0.53), and decreasing MDA by 81% (synergy score 0.54) compared with the APAP group (Table 1). 

**Table 1.** Effects of Decoction Extracts of *Chrysanthemi flos *(CF), *Cassiae semen *(CS), *Lycii fructus* (LF), and Medicine-Food Homology formula (MFHF) and Its *Lactiplantibacillus plantarum* Fermented Products (MFHFF) on Antioxidant Activity ^a, b^

Decoction Extracts	Concentration (µg/mL)	Observed Values (%)	Expected Value (%) ^c^	Synergy ^d^
**DPPH radical scavenging activity**				
CF	45	-9.51 ± 2.22 ^A^	-	-
CS	300	47.66 ± 4.98 ^B^	-	-
LF	150	-25.64 ± 1.52 ^C^	-	-
MFHF	495	62.43 ± 2.06 ^D^	12.51 ± 7.23	4.99
MFHFF	495	61.66 ± 4.39 ^D^	-	-
**Potassium ferricyanide reducing antioxidant power assay-reducing power**				
CF	90	9.94 ± 0.53 ^A^	-	-
CS	600	26.38 ± 1.42 ^B^	-	-
LF	300	11.96 ± 0.78 ^C^	-	-
MFHF	990	40.69 ± 1.20 ^D^	48.27 ± 1.63	0.84
MFHFF	990	46.05 ± 4.30 ^D^	-	-

^a^ Values are expressed as mean ± SD from three independent experiments.

^b^ Different superscripted capital letters indicate significant differences (P < 0.05).

^c^ Expected value is the sum of the observed values from the decoction extracts of the CF, CS, and LF groups.

^d^ Synergy is calculated using the observed value of MFHF / expected value of MFHF, for which a value greater than 1.0 is synergistic, a value of 0.5 - 1.0 is additive, and a value less than 0.5 is antagonistic.

### 4.4. Antioxidant Activity of Decoction Extracts of Chrysanthemi flos, Cassiae semen, Lycii fructus, Medicine-Food Homology formula, and Lactiplantibacillus plantarum Fermented Products

Decoction extracts of CF, CS, and LF (3:20:10 ratio, same as MFHF) were tested for antioxidant activity using DPPH radical scavenging and PFRAP assays. Only CS showed DPPH activity (28.94% inhibition), while CF and LF were inactive. MFHF exhibited significantly higher DPPH inhibition than expected from individual sums (synergy score 4.99) (Table 2). In PFRAP, CS had the highest ferric reducing power; MFHF showed an additive effect (synergy score 0.84) despite being lower than expected (Table 2). Notably, DPPH assay samples were diluted because of strong scavenging activity to ensure accurate measurement. MFHF and MFHFF showed no significant differences in antioxidant activities (Table 2). 

**Table 2.** Effects of Decoction Extracts of *Chrysanthemi flos* (CF), *Cassiae semen* (CS), *Lycii fructus* (LF), and Medicine-Food Homology Formula (MFHF) and its *Lactiplantibacillus plantarum* Fermented Products (MFHFF) on Cell Viability, Glutathione Content, and Malondialdehyde Level in Acetaminophen (APAP)-Treated HepG2 Cells ^a, b^

Variables	Concentration (µg/mL)	Compared with the APAP Group	Observed Values	Expected Value ^c^	Synergy ^d^
**Cell viability**					
APAP	-	100 ± 0 ^D^	-	-	-
CF	22.7	100 ± 4 ^D^	0 ± 4 ^C^	-	-
CS	151.5	153 ± 2 ^B^	53 ± 2 ^B^	-	-
LF	75.8	133 ± 8 ^C^	33 ± 8 ^C^	-	-
MFHF	250	203 ± 15 ^A^	103 ± 15 ^A^	85 ± 3	1.21
**Intracellular glutathione content**					
APAP	-	100 ± 0 ^C^	-	-	-
CF	22.7	137 ± 8 ^B^	37 ± 8 ^B^	-	-
CS	151.5	154 ± 9 ^A, B^	54 ± 9 ^A, B^	-	-
LF	75.8	142 ± 7 ^B^	42 ± 7 ^B^	-	-
MFHF	250	177 ± 12 ^A^	77 ± 12 ^A^	134 ± 12	0.53
**Intracellular malondialdehyde level**					
APAP	-	100 ± 0 ^A^	-	-	-
CF	22.7	63 ± 4 ^B^	-37 ± 4 ^A^	-	-
CS	151.5	24 ± 3 ^C^	-76 ± 3 ^B^	-	-
LF	75.8	64 ± 6 ^B^	-36 ± 6 ^A^	-	-
MFHF	250	19 ± 3 ^C^	-81 ± 3 ^B^	-149 ± 9	0.54

^a^ Values are expressed as mean ± SD from three independent experiments and are normalized to the APAP group (set at 100%).

^b^ Different superscript capital letters indicate significant differences (P < 0.05).

^c^ The sum of the observed values from the decoction extracts of the CF, CS, and LF groups.

^d^ Synergy is calculated using the observed value of MFHF/expected value of MFHF, for which a value greater than 1.0 is synergistic, a value of 0.5 - 1.0 is additive, and a value less than 0.5 is antagonistic.

## 5. Discussion

This study evaluated the hepatoprotective and antioxidant effects of MFHF and its MFHFF. Fermentation preserved key phytochemicals (chlorogenic acid and chrysophanol) and maintained similar antioxidant and cytoprotective activities while promoting probiotic growth. Both formulations improved HepG2 cell viability, restored intracellular GSH, and reduced MDA under APAP-induced oxidative stress. *L. plantarum* fermentation of MFHF showed typical lactic acid bacteria kinetics, including an initial pH drop and a stationary phase after 96 h (8). The higher bacterial count in 5% MFHF compared with a sugar-matched control indicated that MFHF contains additional growth-promoting compounds beyond simple carbohydrates, possibly phenolics or oligosaccharides, consistent with the concept of a prebiotic substrate (9), thus supporting its prebiotic potential. Fermentation did not significantly alter chlorogenic acid or chrysophanol content, indicating that both compounds were chemically stable under the tested conditions. The lack of degradation implies that strain *L. plantarum* BCRC12251 likely exhibits low cinnamoyl esterase and β-glucosidase activity under acidic conditions (pH 3.5 - 5.0), which limits the hydrolysis of phenolic esters or glycosides (10). This interpretation is consistent with a previous report that these enzymes are active only at pH 4.5 - 7.5 (11). New peaks appearing post-fermentation may represent transformed minor metabolites, but the main quality markers remained intact. Overall, chlorogenic acid and chrysophanol can serve as reliable quality-control markers for both MFHF and MFHFF. HepG2 cells are commonly used to evaluate hepatotoxicity owing to their stable hepatic functions and ease of culture (12). Herein, APAP decreased cell viability and intracellular GSH levels while increasing MDA accumulation in HepG2 cells, consistent with previous findings (13). The APAP toxicity involves NAPQI formation, depletion of intracellular GSH, mitochondrial dysfunction, and lipid peroxidation (14). Both MFHF and MFHFF attenuated oxidative injury by restoring cellular redox balance in HepG2 cells, as reflected by increased viability and GSH levels and reduced MDA accumulation. Previous studies have reported hepatoprotective effects of CF in APAP-induced oxidative stress in vivo models and cytoprotective effects of CS and LF in other oxidative stress systems; however, to the best of our knowledge, these extracts have not been specifically examined in APAP-treated HepG2 cells. The present findings extend current knowledge by demonstrating the combined protective effects of CF, CS, and LF in this model. The observed synergistic and additive effects suggested complementary interactions among the herbs. Comparable activities between MFHF and MFHFF indicated that fermentation preserved, rather than enhanced, bioactive efficacy under the tested conditions. This study has some limitations. First, the hepatoprotective effects were evaluated solely in an in vitro HepG2 cell model, which cannot fully reflect the complex metabolic and immunological environment of the liver in vivo. Second, although fermentation did not appear to enhance MFHF’s protective effects, the underlying reasons remain to be clarified. The enzymatic activities of *L. plantarum* (BCRC12251), particularly β-glucosidase and cinnamoyl esterase, were not directly assessed, limiting the interpretation of the biochemical stability of the bioactive compounds after fermentation. Finally, the phytochemical profiling focused on two compounds, and other metabolites or transformation products were not examined. Future work should therefore include mechanistic studies, broader metabolomic profiling, and in vivo validation to clarify how fermentation modulates the bioactivity of MFHF.

Overall, this study provides the first comparative evaluation of MFHF and MFHFF in an APAP-induced HepG2 cell model. Both preparations exhibited hepatoprotection and antioxidant activity by improving cell viability, restoring intracellular GSH levels, and reducing MDA accumulation. Fermentation preserved functional properties and key phytochemical markers. MFHF also exhibited synergistic or additive interactions that support its traditional multi-herb application. These findings suggest that MFHF and MFHFF may protect against oxidative damage through reinforcement of intracellular antioxidant defenses.

## Data Availability

The dataset presented in the study is available upon request from the corresponding author during submission or after publication.
